# Blood collection technique, anticoagulants and storing temperature have minor effects on the isolation of polymorphonuclear neutrophils

**DOI:** 10.1038/s41598-020-71500-1

**Published:** 2020-09-04

**Authors:** Julia Krabbe, Viktor Beilmann, Hanif Alamzad-Krabbe, Svenja Böll, Anke Seifert, Nadine Ruske, Thomas Kraus, Christian Martin

**Affiliations:** 1 Institute of Occupational, Social and Environmental Medicine, Medical Faculty, RWTH Aachen University, Pauwelsstraße 30, 52074 Aachen, Germany; 2 Institute of Pharmacology and Toxicology, Medical Faculty, RWTH Aachen University, Wendlingweg 2, 52074 Aachen, Germany; 3 Department of Pediatrics, Medical Faculty, RWTH Aachen University, University Hospital Aachen, Pauwelstraße 30, 52074 Aachen, Germany

**Keywords:** Experimental models of disease, Cell death, Molecular medicine

## Abstract

In the isolation of polymorphonuclear neutrophils (PMNs) the technique and other external factors can have great influence on the quality and quantity of isolated neutrophils. To elucidate the influence of the blood collection technique, anticoagulants and storing temperature on isolated PMNs healthy volunteers provided blood samples with different needles and collection techniques, anticoagulants (EDTA, heparin, citrate) and storing temperatures (4, 22, 37 °C). From each blood sample PMNs were isolated and compared regarding number of PMNs and oxidative burst. The blood collection technique, anticoagulants and storing temperature had minor impact on isolated PMNs. All three tested cannulas and anticoagulants can be used to obtain blood samples for PMN isolation. For storing temperatures 37 °C should be preferred. Regarding time between the PMN isolation and the actual experiments, a time span of maximum 1 h should be targeted.

## Introduction

Isolated polymorphonuclear neutrophils (PMNs) can be used in multiple consecutive experiments depending on the scientific question^1–3^. Especially investigations of PMN functions, e.g. respiratory burst activity, phagocytosis, transmigration or microbial killing, require reliable isolation techniques. Ideally, the isolated sample would contain all PMNs that were present in the whole blood with no further activation of these cells. However, PMNs are, as first responders of the immune system, very fragile and easily activated. In an inactivated state their half-life is approximately 7 h^4–6^ and they are influenced by the circadian rhythm^7,8^ with an increase of PMNs in plasma over the day. Thus, the isolation technique and other external factors can have great influence on the quality and quantity of isolated neutrophils. Interestingly, most manuscripts describing PMN isolation procedures do not mention the specific collection technique used to obtain blood for PMN isolation^2,3,9–11^. Only a few mention the technique but with few details^12–14^.

Generally, isolated cells could be influenced by disturbed flow, shear stress and other turbulences during the blood collection. PMNs can adhere to the vessel walls and migrate into the target tissue under physiological flow conditions. However, shear stress can lead to enhanced adhesion among PMNs^15^, which is useful for transmigration but unfavourable for cell isolation without activation. It has been hypothesised that PMNs have fluid shear stress sensors which can control PMN activation^16^. Furthermore, exposure to fluid shear stress can lead to leukocyte membrane disruption^17^. Best to our knowledge, it has not been sufficiently investigated if and to what degree blood collection could cause shear stress to the collected samples. Consecutively, the hypothesis of this study was that the blood collection technique regarding collection with negative pressure aspiration versus free flow, as well as regarding the inner diameter of the cannula influences the activation of PMNs and lead to reduced numbers of PMNs. Additionally, the used coagulants and storing temperatures of isolated PMNS could also considerably influence the isolated PMNs in number and function.

To elucidate the influence of the blood collection technique on quality and quantity of isolated PMNs 10 healthy volunteers provided 5 different blood samples (10 ml each): (1) blood obtained with a 21 gauge (G) butterfly vein cannula with free flow into a sample vessel (BF-FF), (2) blood obtained with a 21G butterfly vein cannula drawn with a 10 ml syringe (BF-S), (3) blood obtained with a 18G vein catheter with free flow into a sample vessel (VC-FF) (4) blood obtained with a 18G vein catheter drawn with a 10 ml syringe (VC-S) and (5) blood obtained with a 22G vacuum cannula into an vacuum vessel (V-V). From each blood sample PMNs were isolated and compared regarding amount of PMNs and respiratory burst activity. Additionally, chemokine levels of the PMN supernatants were determined from blood samples of 4 volunteers. For comparison of anti-coagulants and storing temperatures, 6 volunteers donated blood which was mixed with ethylenediaminetetraacetic acid (EDTA), heparin or sodium citrate, respectively. From those blood samples PMNs were isolated and either immediately analysed or stored at 4, 22 or 37 °C for 3 h.

## Results

**Figure 1:**
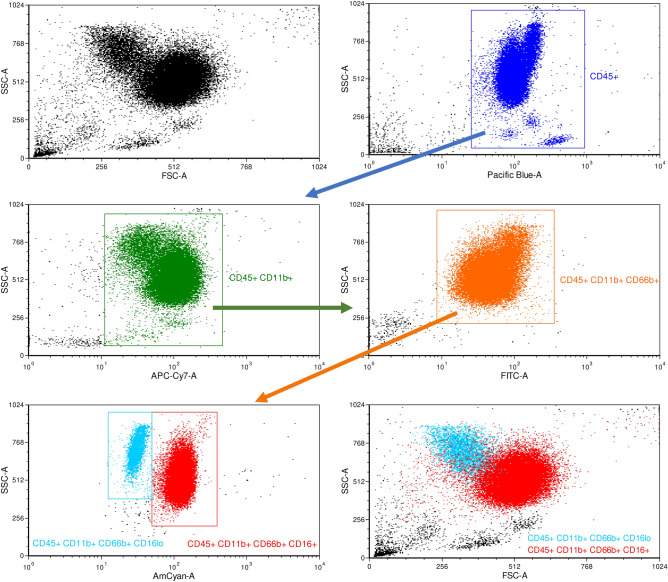
Specific PMN staining on isolated cells: PMNs were identified with the following gating strategy: after identification of leukocytes via CD45 positive selection (blue gate), cells were declared as PMNs that also expressed CD11b (green gate), CD66b (orange gate) and CD16 (turquoise gate for low positive expression and red gate for positive expression). X-axis: Side-scatter area (SSC-A), y-axis fluorescence of indicated antibody (CD66b—FITC, CD11b—APC-Cy7, CD45—VioBlue, CD16—VioGreen and CD62L—PE-Cy7).

To assess the quality of isolated samples the isolated PMNs were specifically stained and analysed by flow cytometry. PMNs were identified by expression of the following markers: cluster of differentiation (CD) 45, CD11b, CD66b and CD16^12^. Regarding CD16, two positive populations could be observed, one with low positive and one with higher expression of CD16 (Fig. 1 Panel CD45+ CD11b+ CD66b+ CD16+). The population with weaker expression of CD16 corresponds to apoptotic PMNs^18^ indicating activation or damaging and consecutive induction of apoptosis in PMNs during the isolation procedure. 94.4% ± 1.2 (SD) of the isolated cells were CD45+ CD11b+ CD66b+ CD16+ cells indicating a successful isolation procedure of PMNs. An average yield of between 6.3 and 4.9 × 10^5^ cells/ml was achieved. All blood donors had plasma leukocyte and PMN levels within a physiological range.

**Figure 2:**
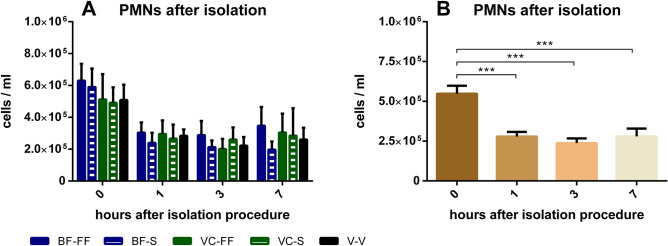
Effects of blood collection technique and storing time after isolation on PMN count: PMNs were counted 0, 1, 3 and 7 h after the isolation process. (**A**) PMN counts categorised by collection technique: BF-FF, butterfly cannula with free flow; BF-S, butterfly cannula with syringe; VC-FF, vein catheter with free flow; VC-S, vein catheter with syringe; V-V, vacuum cannula. (**B**) PMN counts depicted for all methods together. ***p < 0.001, BF-FF and V-V n = 10, BF-S, VC-FF and VC-S n = 9, data are shown as mean ± SEM**.**

The blood collection technique seemed to have no effect on the number of isolated PMNs for all storing times (Fig. 2A). Already 1 h storing in a water bath at 37 °C after the isolation procedure caused a significant decrease in PMN counts. After that the number of PMNs remained stable, however, only half of the initially isolated PMNs were present (Fig. 2B).

**Figure 3:**
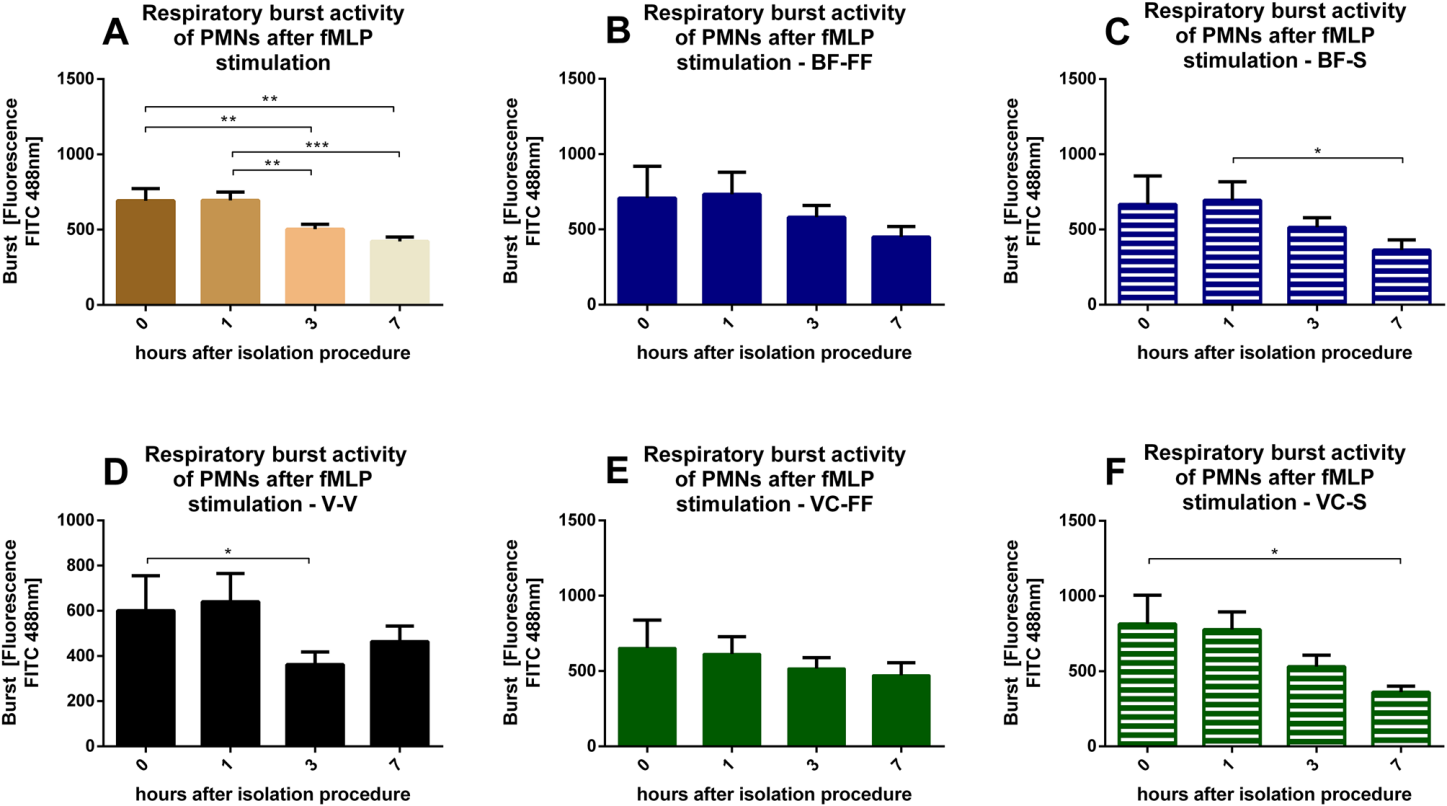
Effects of storing time and blood collection technique on PMN respiratory burst activity: Fluorescence as indicator of accumulation of reactive oxygen species (oxidative burst) was determined, depicted is the mean over 1,000 cells counted. (**A**) Respiratory burst activity depicted for all methods 0, 1, 3 and 7 h after the isolation process. (**B**) Respiratory burst activity for different storing times drawn with butterfly cannula in free flow. (**C**) Respiratory burst activity for different storing times drawn with butterfly cannula and syringe. (**D**) Respiratory burst activity for different storing times drawn with vacuum cannula. (**E**) Respiratory burst activity for different storing times drawn with vein catheter in free flow. (**F**) Respiratory burst activity for different storing times drawn with vein catheter with syringe. *p < 0.05; **p < 0.01, ***p < 0.001, BF-FF and V-V n = 10, BF-S, VC-FF and VC-S n = 9, data are shown as mean ± SEM**.**

The respiratory burst activity of PMNs is significantly reduced after the 3- and 7-h storing period, whereas after 1 h no changes could be observed (Fig. 3A). In general, there is a trend towards decreased number of PMNs over time. In detail, time point 3 h is significantly different for V-V (0 h vs. 3 h, Fig. 3D), as well as time point 5 h for BF-S (1 h vs. 7 h, Fig. 3C) and VC-S (= 0 vs. 7 h, Fig. 3F). When we compare free flow (FF) versus syringe (S) for the butterfly and the venous cannula the decrease in respiratory burst seem to be more prominent when syringes are used (Fig. 3B vs. C and Fig. 3E vs. F).

To analyse the potential of activation of PMNs differences of *N*-Formylmethionine-leucyl-phenylalanine (fMLP) stimulated PMNs and unstimulated PMNs (PBS) were determined by fluorescence in flow cytometry.

**Figure 4:**
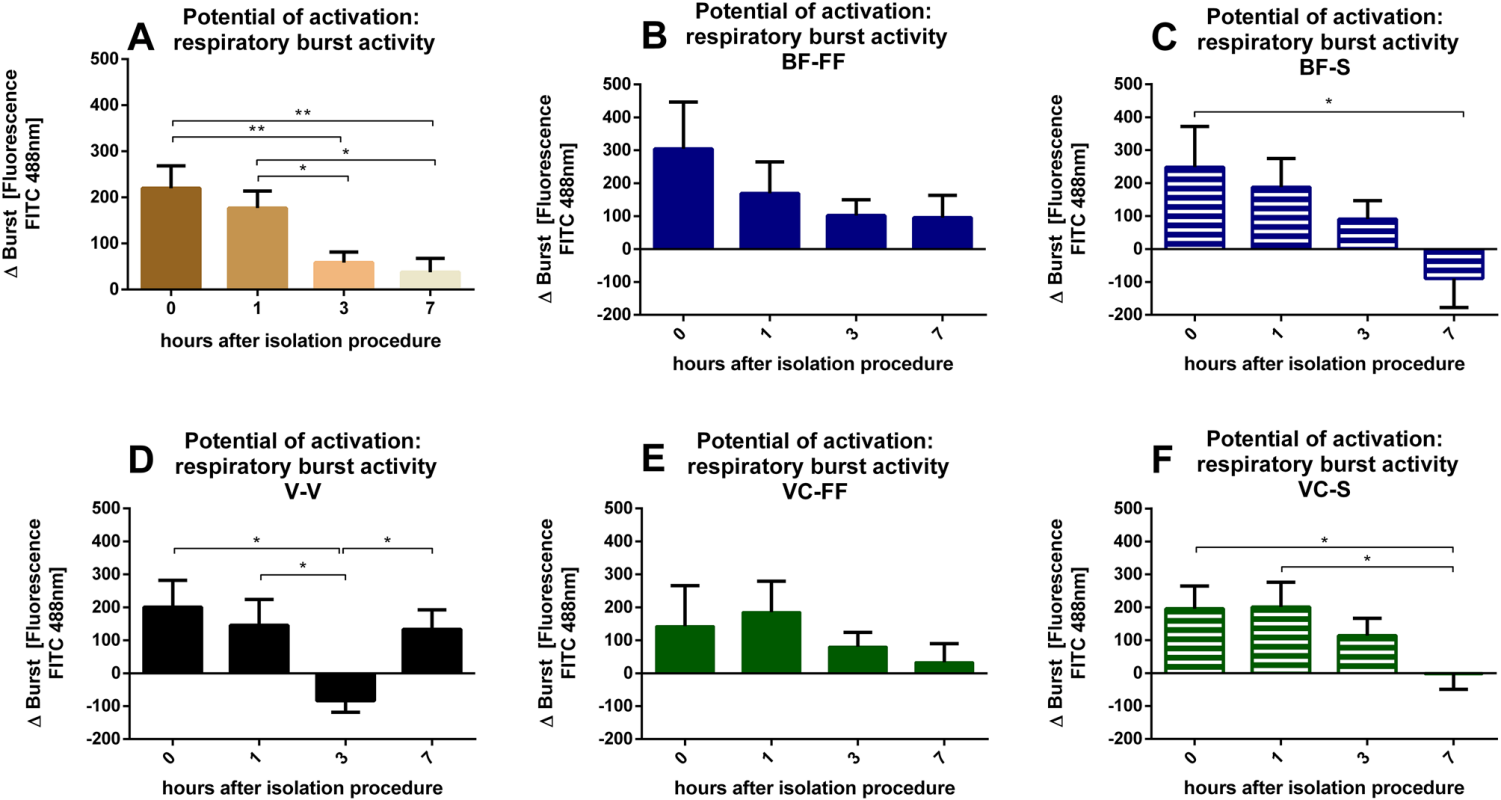
Effects of storing time and blood collection technique on PMN burst—potential of activation: Depicted is the difference between the mean fluorescence of the activated sample and the mean fluorescence of the control sample. Fluorescence was used as indicator of accumulation of reactive oxygen species (oxidative burst). (**A**) Δ Burst depicted for all methods 0, 1, 3 and 7 h after the isolation process. (**B**) Δ Burst for different storing times drawn with butterfly cannula in free flow. (**C**) Δ Burst for different storing times drawn with butterfly cannula and syringe. (**D**) Δ Burst for different storing times drawn with vacuum cannula. (**E**) Δ Burst for different storing times drawn with vein catheter in free flow. (**F**) Δ Burst for different storing times drawn with vein catheter with syringe. *p < 0.05; **p < 0.01; ***p < 0.001, BF-FF and V-V n = 10, BF-S, VC-FF and VC-S n = 9, data are shown as mean ± SEM.

Similar to the general respiratory burst activity of the isolated PMN, a significant reduction after 3 and 7 h of storing period was observed for comparison of stimulated vs. basal release of oxygen radicals (Fig. 4A). Only the free flow blood collection technique showed no significant decrease in the delta of activity, even a trend seems to be clear (Fig. 4B, E): While there were no differences present for BF-FF (Fig. 4B), for BF-S significant differences in respiratory burst activity could be observed between 0 and 7 h (Fig. 4C). V-V showed differences between 3 h and the other time points (Fig. 4D). There were no differences present for VC-FF (Fig. 4E) and for VC-S significant differences in respiratory burst activity could be observed between 0 and 7 h and 1 and 7 h (Fig. 4F).

Blood collection technique had no effect on chemokine levels of isolated PMNs at all storing periods. However, significant differences in chemokines between different storing periods could be observed (Suppl. Figure 1).

**Figure 5:**
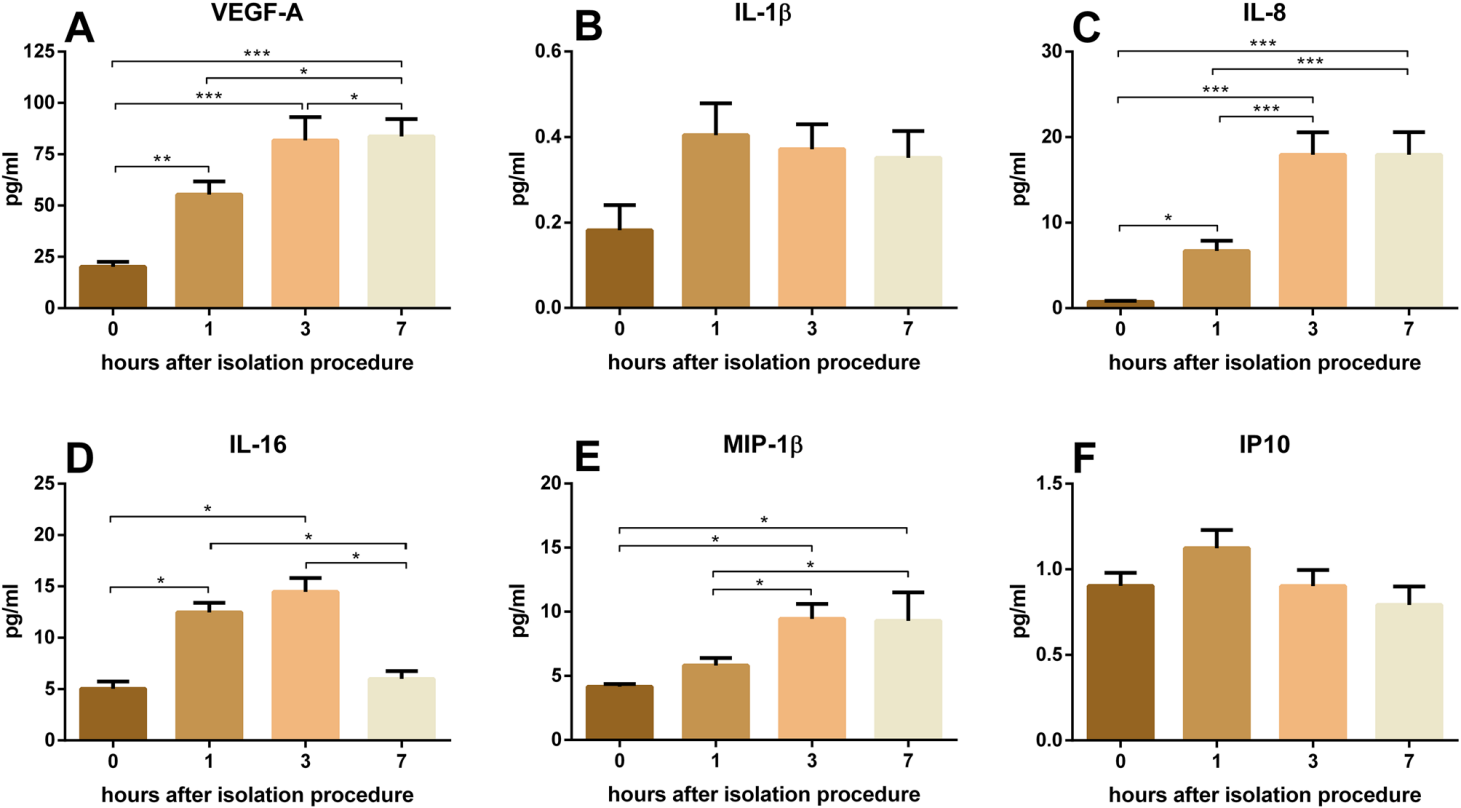
Effects of storing time on chemokine levels: (**A**) VEGF-A levels for different storing times. (**B**) IL-1β levels for different storing times. (**C**) IL-8 levels for different storing times. (**D**) IL-16 levels for different storing times. (**E**) MIP-1β levels for different storing times. (**F**) IP-10 levels for different storing times. *p < 0.05; **p < 0.01; ***p < 0.001, BF-FF and V-V n = 10, BF-S, VC-FF and VC-S n = 9, data are shown as mean ± SEM.

Vascular endothelial growth factor A (VEGF-A), interleukin (IL)-8 and macrophage inflammatory protein 1 (MIP-1) β levels rose continuously over 7 h (Fig. 5A, C, E) or 3 h only for IL-16 (Fig. 5D). IL-16 levels decreased afterwards (7-h time point, Fig. 5D). The chemokine release for IL-1 β and Interferon-gamma induced protein 10 (IP10, Fig. 5B, F) were relatively constant over time. Granulocyte–macrophage colony-stimulating factor (GM-CSF), IL-5, IL-7, IL-12/IL-23p40, IL-15, IL-17A, tumor necrosis factor (TNF)-β, interferon gamma (IFN-γ), IL-2, IL-4, IL-6, IL-10, IL-12p70, IL-13 and TNF-α levels were under the detection limit, while levels of eotaxin, eotaxin-3, thymus and activation regulated chemokine (TARC), MIP-1α, monocyte chemotactic protein (MCP)-1, macrophage-derived chemokine (MDC), MCP-4 and IL-1α showed no alterations of levels for different storing times and blood collection techniques (Suppl. Figure 1).

**Figure 6:**
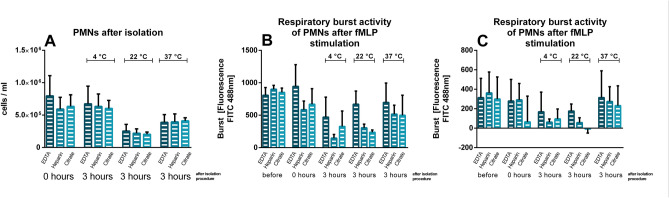
Effects of anticoagulant for different storing temperatures after isolation: blood samples were mixed with EDTA, heparin or sodium citrate. (**A**) PMN counts. (**B**) respiratory burst activity, determined by fluorescence as mean over 1,000 cells counted. (**C**) Δ Burst as difference between the mean fluorescence of the activated sample and the mean fluorescence of the control sample. No significant differences between the anticoagulants were found. n = 6 except for the timepoint ‘before’ (n = 3), data are shown as mean ± SEM.

For all three different anticoagulants (EDTA, heparin and sodium citrate) and storing temperatures, no significant differences could be detected for PMN counts (Fig. 6A), respiratory burst activity (Fig. 6B), or potential of activation (Fig. 6C). In addition, no differences occurred for respiratory burst activity and potential of activation for the comparison of the whole blood samples before the isolation and after (Fig. 6B + C).

**Figure 7:**
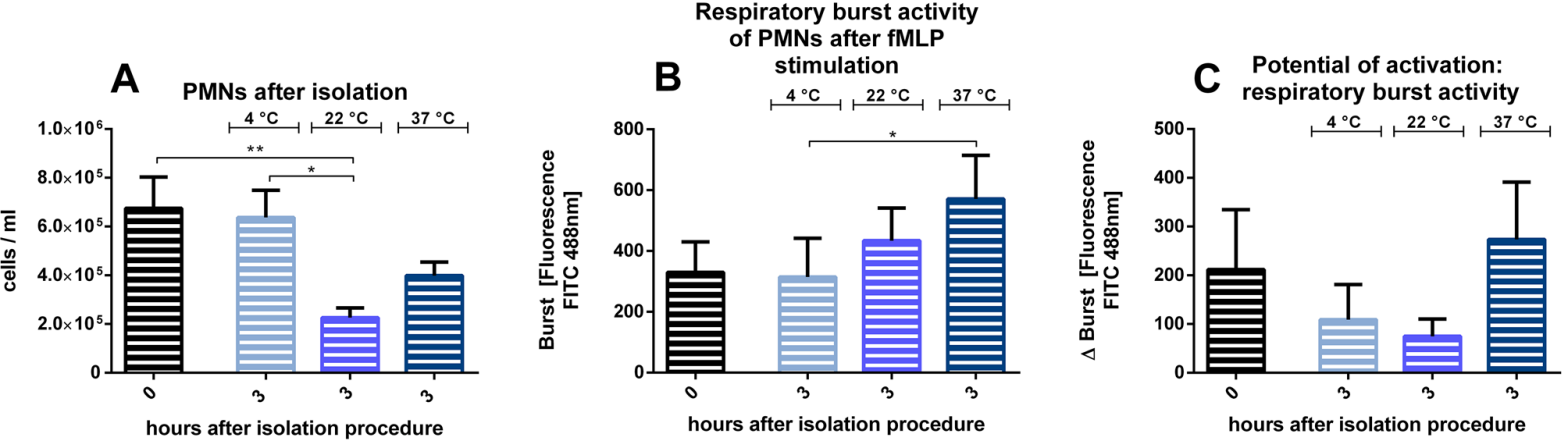
Effects of storing temperatures after isolation for all coagulants combined: isolated PMNs were stored at 4, 22 or 37 °C for 3 h. (**A**) PMN counts. (**B**) respiratory burst activity, determined by fluorescence as mean over 1,000 cells counted. (**C**) Δ Burst as difference between the mean fluorescence of the activated sample and the mean fluorescence of the control sample. *p < 0.05; **p < 0.01, n = 6, data are shown as mean ± SEM.

Surprisingly, the storing temperatures at 22 °C (compared to after the isolation and to 4 °C) showed a significant decrease in PMN counts after 3 h (Fig. 7A). Furthermore, for PMNs stored at 37 °C a significantly higher respiratory burst activity compared to 4 °C could be observed (Fig. 7B). Although tendencies of decreased potential of activation could be seen for 4 and 22 °C, no significant differences could be detected (Fig. 7C).

## Discussion

Best to our knowledge, this is the first study to determine the role and possible effects exerted by the blood collection technique on isolated PMN samples. The blood collection technique had little effects on the number of isolated PMNs for all storing times. However, the storing time had great influence on number of PMNs, respiratory burst activity and chemokine levels. Additionally, anticoagulants and storing temperature were examined, showing only minor effects of the storing temperature regarding PMN count.

PMN isolation procedures are often used to study the cellular immune responses in biomedical research. The hypothesis was that blood collection techniques including collection with negative pressure aspiration due to vacuum containers and syringes activate PMNs during the collection process which could lead to a reduced PMN number. Additionally, the inner diameter may influence the disturbances and consecutively activate PMNs. Respiratory burst activity and potential of activation were influenced by techniques using aspiration or negative pressure but did not depend on inner diameter of the cannula. Under physiological conditions in a water bath at 37 °C, the storing period (time after isolation) had considerable effects on all examined characteristics of the isolated PMNs.

In literature, a typical storing time up to 8 h of after the isolation^6^ is reported, which is only partially supported by our data. The number of PMNs is already lowered after 1 h most likely due to activation during the isolation procedure and consecutive apoptosis. After that the number of PMNs remains stable. Another explanation for the loss of PMNs after 1, 3 and 7 h could be adherence of PMNs to tube walls during storage. For the 0 h timepoint, the isolated PMNs were immediately processed, while the PMNs for the other time points were transferred in tubes for storage. This would favour an immediate processing of PMNs after isolation for consecutive experiments. However, respiratory burst activity and potential of activation were not significantly different in whole blood samples determined before PMN isolation compared to stored isolated PMN samples after 3 h. Thus, the adherence to the tubes during storage should be a minor influence.

The chemokine levels of 4 chemokines that increased over storing period are typically released by PMNs^19–21^. This indicates an at least partial activation of the isolated PMNs and the decreasing number of PMNs and respiratory burst activity. IL-16 decreased at the 7 h time point, due to the half-life of 3 h^22^. The IL-16 secretion could have only been active right after the isolation process and IL-16 already disintegrated after 7 h. In conclusion, the ideal timing for the beginning of experiments after the isolation process would be within 1 h after the isolation. Later time points could be accompanied with considerably lower respiratory burst activity potential even though the numbers of PMNs would be stable after the isolation.

Storing conditions could also influence number and activation of PMNs. In this study, storage a water bath at 37 °C (physiological temperature) was explicitly chosen and should prevent effects of cooling and reheating. Other protocols include storage or centrifugation under cooling conditions, e.g. 4 °C to prevent or at least decelerate PMN activation^23^. In addition, storage of isolated PMNs was examined also for 4 °C and 22 °C revealing disadvantages regarding PMN count for 22 °C. Most studies in literature investigated the storing temperatures of obtained blood samples prior to PMN isolation^24–26^ but not after. One study reports that storage at 4 °C with consecutive warming to 37 °C for function tests under physiological conditions can affect and activate the PMNs regarding adherence molecule expression and recommends storage at room temperature^27^. However, we cannot recommend storage at room temperature as PMN count was reduced for storage at 22 °C. In conclusion, based on our findings storage at 37 °C should be preferred rather than storage at lower temperatures if consecutive function tests are also performed at 37 °C. In general, it seems beneficial to not alter the temperature after storing for best PMN count and function.

The anticoagulant used during blood collection had also no effect on PMN count and respiratory burst activity. These findings are in contrast to a study by Freitas and colleagues^28^, which identified EDTA blood samples as most yielding but with less activity after phorbol myristate acetate (PMA) stimulation. However, since no concentrations of anticoagulants were described in the study a direct comparison to our results could be misleading. Differences between EDTA and citrate are less likely than between them and heparin due to the same mechanism of EDTA and citrate inhibiting coagulation via chelating of calcium ions. Heparin has been known to interfere with neutrophils in inhibiting^29,30^ and apoptosis-inducing manners^31^. Furthermore, EDTA has been described to cause pseudo-neutropenia in blood samples due to EDTA-induced clotting of PMNs^32^ , which is an in vitro phenomenon potentially interfering with the PMN isolation process. Citrate has been reported to inhibit fMLP and respiratory burst activity of PMNs^33^.So all three anticoagulants could potentially interfere with PMN isolation and functions, nevertheless we could not detect significant differences between them. Thus, potential differences seem to be minor, and no specific anticoagulant can be recommended or excluded.

In contrast to our hypothesis, the collection technique itself has rather little effects on number, respiratory burst ability or chemokine secretion of the isolated PMNs. As PMNs are known for their fragility and easy to activate, this result is surprising. However, there were no significant differences between the techniques regarding PMN count or chemokine levels. One explanation could be the physiological adaptation of PMNs to the circulation in the human body. In the arterial systemic circulation PMNs are exposed to the systolic blood pressure of approximately 100–140 mmHg under physiological conditions and up to 250 mmHg under exercise or arterial hypertension. Thus, PMNs could be used to sudden pressure changes and even shear stress or disturbed flow while they travel through the capillary system. Additionally, most mechanisms of responses to shear stress or disturbed flow have their origin in effects on the endothelial cells or other blood cells but not PMNs themselves^15^. In this study PMNs are drawn into sample vessels and then immediately isolated. Thus, influences of the endothelium or other blood cells are not present or rather reduced, limiting the influence of those mechanisms on PMNs. Accordingly, in this study even an additional negative pressure combined with a disturbing flow within the cannula did not lead to an additional activation of PMNs.

Interestingly, respiratory burst activity and potential of activation showed significant differences between storing times in techniques including negative pressure—BF-S, V-V and VC-S. This trend indicates even considerable differences in the isolated PMN samples regarding consecutive activation in the experiments, since activation and respiratory burst of PMNs leads to a cascade of processes with amplification mechanisms^33,34^. Thus, this slight advantage of techniques with free blood flow in comparison to techniques including aspiration could improve the quality of the obtained blood samples for PMN isolation. Consequently, if the blood collection for the PMN experiments is focused on respiratory burst activity or activation of PMNs the blood should be drawn with free blood flow.

The techniques including free blood flow can be stressing for the volunteer or patient, which has to be considered. Patients have different vein-sizes and an individual tolerance to pain, duration of the procedure and the sight of blood, potentially complicating the blood collection procedure. Especially the blood collection with free blood flow (BF-FF + VC-FF) was uncomfortable for the volunteers due to the visible blood spill into the sample vessel. Additionally, the vein catheter left a bigger lesion at the sampling point due to its greater calibre, requiring longer pressure on the lesion by the volunteers to prevent haematoma formation. In summary, if healthy volunteers with an expected easy puncture are included as blood donors, blood collection techniques with free blood flow should be preferred. In case of patients with possible difficulties regarding puncture, low calibre cannulas with vacuum or aspiration should be preferred due to fast collection time and small manageable lesions unless blood collection is combined with a medical intervention, e.g. placement of a vein catheter for an infusion.

In conclusion, the blood collection technique, anticoagulants and storing temperatures have only minor impact on the quality and quantity of isolated PMNs. All three tested cannulas—butterfly cannula, vein catheter and vacuum cannula—can be used to obtain blood samples for PMN isolation. If PMN respiratory burst activity is the focus of the conducted experiments collection techniques including free blood flow could be beneficial, if suitable for the subjects or patients. Regarding time between the PMN isolation and the actual experiments, a time span of maximal 1 h may be beneficial, although there were no significant differences in PMN counts between 1 and 7 h. No anticoagulant examined showed specific advantages or disadvantages, thus, all three could be used. Storage of isolated PMNs should be done at 37 °C, while storage at 4 °C could be accompanied by PMN activation during re-warming to 37 °C for PMN function tests. However, most beneficial experiments should start soon after isolation.

## Methods

### Subjects

Thirteen healthy volunteers (9 men and 4 women) were included in this study. The mean age was 29.2 ± 8.9 (SD) years. All participants were healthy and had no prior blood diseases or inflammatory conditions/infections within the last 2 weeks. The study was approved by the ethics committee of the Medical Faculty, RWTH Aachen University (EK 67/19) and was performed in compliance with the Declaration of Helsinki ethical principles for medical research involving human subjects. All volunteers gave written informed consent prior to inclusion.

### Agents and materials

Safety-Multifly vein cannulas (21 gauge (G)) and 2.7 ml S-Monovettes were purchased by Sarstedt (Nümbrecht, Germany), VenflonPro Safety vein catheters (18G), Vacutainer Eclipse vaccum cannulas (22G) and the corresponding vacutainer sample vessels were purchased from Becton Dickinson GmbH (Heidelberg, Germany). B. Braun Omnifix Solo Luer syringes 10 ml were obtained by B. Braun Melsungen AG (Melsungen, Germany). Pancoll human, Density: 1.077 g/ml, was purchased by PAN-Biotech GmbH (Aidenbach, Germany) and sodium citrate 3.13% was purchased by EIFELFANGO Chem. Pharm. Werk GmbH & Co. KG (Bad Neuenahr-Ahrweiler, Germany). Calcium free RPMI 1,640 Medium was purchased from United States Biological (Salem, MA, USA). Dimethylsulfoxid (DMSO), Dihydrorhodamine 123 (DHR) and N-Formylmethionine-leucyl-phenylalanine (fMLP) were purchased from Merck KGaA (Darmstadt, Germany). Antibodies for flow cytometry analysis (CD66b—FITC, CD11b—APC-Cy7, CD45—VioBlue, CD16—VioGreen and CD62L—PE-Cy7) were purchased from eBioscience (Frankfurt am Main, Germany).

### Blood collection

**Figure 8:**
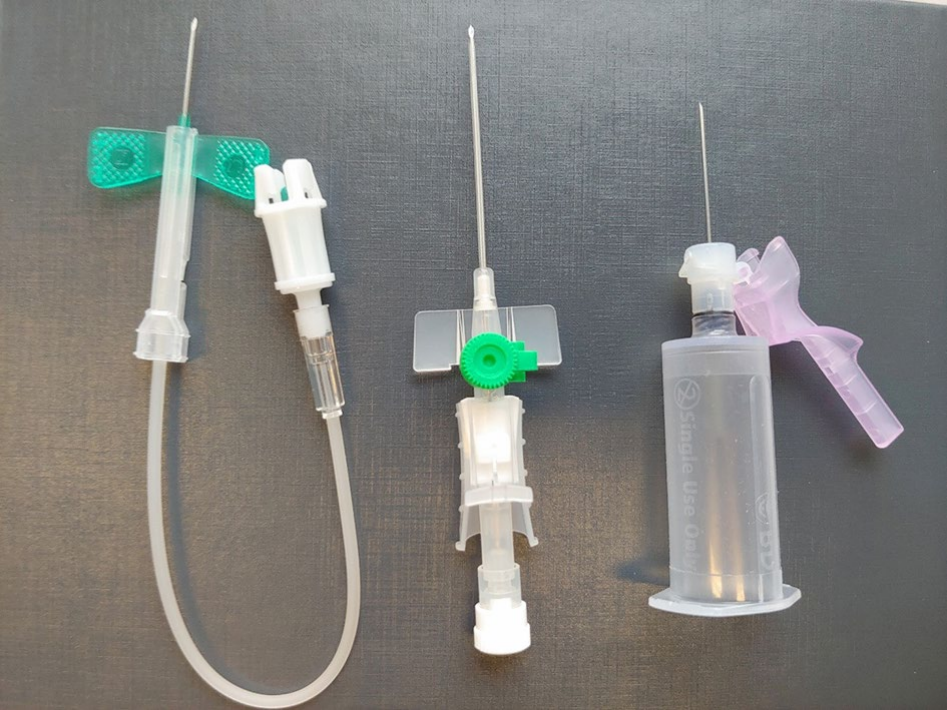
Cannulas used for blood collection: three different cannulas were used: a 18G vein catheter (VC), a 21G butterfly vein cannula (BF) and a 22G vacuum cannula (V). The order of the cannulas used was randomized for each subject.

Blood collection was scheduled for each subject between 7 and 7:30 am to prevent differences due to circadian rhythm. All blood collection procedures were conducted by the same trained medical student under supervision by a physician. Three different cannulas were used: (1) a 21 (G) butterfly vein cannula, (2) a 18G vein catheter and (3) a 22G vacuum cannula (Fig. 8). The order of the different cannulas was randomized for each subject. Five different 10 ml blood samples were obtained from each subject: (1) blood obtained with the butterfly vein cannula with free flow into a sample vessel (BF-FF), (2) blood obtained with the butterfly vein cannula drawn with a 10 ml syringe (BF-S), (3) blood obtained with the vein catheter with free flow into a sample vessel (VC-FF), (4) blood obtained with the vein catheter drawn with a 10 ml syringe (VC-S) and 5) blood obtained with the vacuum cannula into an vacuum vessel (V-V). Additionally, 2.7 ml blood was obtained via the butterfly vein cannula into a S-Monovette with EDTA for a haemogram analysed in the Laboratory Diagnostics Centre (LDC) of the University Hospital of RWTH Aachen University.

For 2 volunteers blood samples were not obtained for all collection techniques. One subject moved during the switch between free flow and syringe with the butterfly cannula (BF-S) leading to dislocation of the cannula and abort of the blood collection. For a second subject the puncture with the vein catheter (VC-FF and VC-S) was not successful. However, for each technique at least 9 blood samples were analysed according to power analysis.

For comparison of anticoagulants and storing temperatures, 6 volunteers provided blood samples. The blood collection was performed with the butterfly vein cannula drawn with a 10 ml syringe pre-filled with sodium citrate (0.31%), EDTA (0.16%) or heparin (40 IU/ml).

### Isolation of PMNs

Each blood sample was immediately transferred into a vessel filled with 5 ml phosphate buffered saline (PBS) and carefully inverted to mix the components. Afterwards the blood mixture was slowly layered onto Pancoll without mixing of the two layers. After centrifugation for 30 min at 20 °C with 300 G, the supernatants were removed and the pellet was resuspended in 10 ml erythrocyte lysis buffer (4.145 g NH_4_Cl_2_, 0.5 g KHCO_3_, 0.02 g EDTA, 500 ml aqua dest.) for 5 min with immediate PBS addition and centrifugation for 5 min at 20 °C with 300 G afterwards. This step was repeated once more. The obtained cell pellet was then resuspended in calcium free RPMI medium, split into 4 vessels and stored at 37 °C in a water bath for 1, 3 or 7 h until further analysis. The sample for 0 h after the isolation was immediately analysed.

For comparison of anticoagulants and storing temperatures, the isolated PMNs were either immediately analysed or stored at 4, 22 or 37 °C for 3 h.

### Determination of the number of PMNs

From each blood sample for each time point 250 µl of the cell suspension was used for cell counting with the BD LSR Fortessa analyzer (BD Bioscience, Germany). Events were counted for three minutes. Afterwards the PMN count was calculated using the number of events identified by size (via forward scatter, FSC) and granularity (via side scatter, SSC) as PMNs, the flow rate and the sample volume. Analysis was conducted with FCS Express 4 Flow Research Edition (De Novo Software, Glendale, CA, USA).

### Quality of PMN isolation

To assess the quality of isolated samples the isolated PMNs of 3 volunteers were stained and analysed by flow cytometry. The cell pellet was resuspended in 1 ml PBS and the staining solution containing specific antibodies was added: CD66b—FITC (1:10), CD11b—APC-Cy7 (1:25), CD45—VioBlue (1:10), CD16—VioGreen (1:10) and CD62L—PE-Cy7 (1:25). After the staining for 45 min in darkness at 4 °C, 1 ml PBS was added and the samples were centrifuged for 5 min at 4 °C with 300 G. This step was repeated once more. Afterwards, the cell pellet was resuspended in 250 µl PBS and used for flow cytometry with the BD LSR Fortessa analyzer (BD Bioscience, Germany). A minimum of 10,000 events were collected for evaluation.

### Respiratory burst activity of PMNs

From each blood sample for each time period 250 µl of the cell suspension was used for respiratory burst induction with activation or without as control. Additionally, for 3 volunteers whole blood samples were obtained and 250 µl were used. The cells suspension was given into 250 µl PBS, 15 mM Dihydrorhodamine 123 (DHR), and 10 µM fMLP for activation or PBS as control was added. The samples were incubated at 37 °C for 20 min, the reaction was then stopped by putting the tubes on ice for 10 min. Afterwards, the samples were centrifuged at 4 °C for 5 min with 1,000 G. The supernatant was discarded, the cell pellet resuspended and analysed with the BD LSR Fortessa analyzer (BD Bioscience, Germany). Accumulation of reactive oxygen metabolites, e.g. superoxide anions, hydrogen peroxide or hypochlorous acid was measured via fluorescence measurement caused by enzymatic oxidation of DHR and captured for 3 min at an excitation of 488 nm and emission of 520 nm. The mean fluorescence was determined for activated cells and control. Analysis was conducted with FACS Express 4 Flow Research Edition (De Novo Software, Glendale, CA, USA).

### Cytokine and chemokine levels

From each blood sample for each time point 250 µl of the cell suspension was centrifuged at 4 °C for 5 min with 1,000 G. The supernatant was collected and frozen for cytokine and chemokine determination at − 80 °C. Levels of IL-1β, IL-2, IL-4, IL-6, IL-8, IL10, IL-12p70, IL-13, TNF-α, and IFN-γ (V-PLEX Proinflammatory Panel 1 Human Kit), Eotaxin, Eotaxin-3, IL-8, IL-8 (HA), IP-10, MCP-1, MCP-4, MDC, MIP-1α, MIP-1β, and TARC (V-PLEX Chemokine Panel 1 Human Kit) and GM-CSF, IL-1α, IL-5, IL-7, IL-12/IL-23p40, IL-15, IL-16, IL-17A, TNF-β, and VEGF-A (V-PLEX Cytokine Panel 1 Human Kit) were analysed via an electrochemiluminescent immunoassay according to the manufacturer’s instructions (Meso Scale Discovery (MSD), Gaithersburg, MD, USA) using the Meso Quick Plex SQ 120. Raw data were analysed using the Discovery Workbench 4.0 software (MSD).

### Statistics

Based on own preliminary data, the experiments were planned with a statistical power of 80% and an alpha error of 0.05 (corrected for multiple comparisons) in order to detect differences between the isolated PMNs greater than 25,000 cells/ml (G*Power v. 3.1.9.2, Düsseldorf, Germany), which resulted in a group size of at least n = 9. To account for possible difficulties with the blood collection 10 volunteers were recruited.

For the comparison of anticoagulants and storing temperatures, differences greater than 35,000 cells/ml should be detected resulting in a group size of at least n = 6. Since only one blood collection technique was used, 6 volunteers were recruited.

Data analysis was performed using SAS 9.4 (SAS Institute Inc., Cary, North Carolina, USA). All data are shown as mean ± SEM and n indicates the number of blood donors.

Q-Q residual plots and row histograms were used to observe the data distribution. Analysis was carried out using general linear mixed model analysis (Proc Glimmix; SAS software V 9.4) assuming a normal distribution. In case of heteroscedasticity, the df were adjusted by the Kenward–Rogers method. P values were always adjusted by the simulated-Shaffer procedure. Data were plotted with GraphPad Prism software V 6 (GraphPad Software Inc., USA). Analysis differences were assumed to be significant with *p* < 0.05.

## Supplementary information

Supplementary information

## Data Availability

The datasets generated during and/or analysed during the current study are available from the corresponding author on reasonable request.
