# Supplementary material for: Blood collection technique, anticoagulants and storing temperature have minor effects on the isolation of polymorphonuclear neutrophils
Source: Sci Rep. 2020 Sep 4;10:14646. doi: 10.1038/s41598-020-71500-1 (PMC7474093; doi:10.1038/s41598-020-71500-1)
Supplement: Supplementary file 1 — Supplementary information [file 41598_2020_71500_MOESM1_ESM.pdf]

Supplemental Figure 1

Blood collection technique, anticoagulants and storing temperature have minor effects on the isolation of polymorphonuclear neutrophils

Julia Krabbe[1], Viktor Beilmann[1/2], Hanif Alamzad-Krabbe[2], Svenja Böll[2/3], Anke Seifert[2], Nadine Ruske[2], Thomas Kraus[1] and Christian Martin[2]

[1]Institute of Occupational, Social and Environmental Medicine, Medical Faculty, RWTH Aachen University, Pauwelsstraße 30, 52074 Aachen, Germany

[2]Institute of Pharmacology and Toxicology, Medical Faculty, RWTH Aachen University, Wendlingweg 2, 52074 Aachen, Germany

[3]Department of Pediatrics, Medical Faculty, RWTH Aachen University, University Hospital Aachen, Pauwelstraße 30, 52074 Aachen, Germany

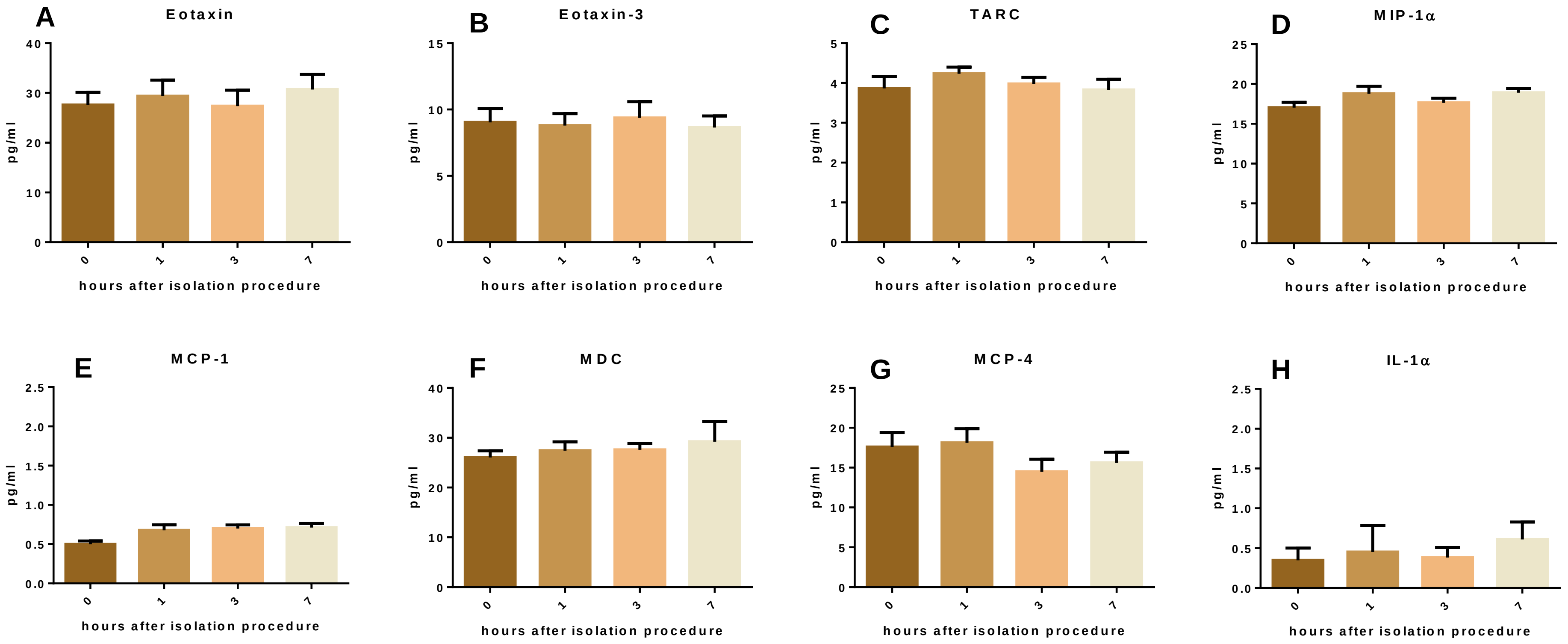

Supplemental Fig. 1: ***Effects of storing time after isolation on chemokine levels:*** **(A)** eotaxin, **(B)** eotaxin-3, **(C)** TARC, **(D)** MIP-1α, **(E)** MCP-1, **(F)** MDC, **(G)** MCP-4 and **(H)** IL-1α. BF-FF & V-V n=10, BF-S, VC-FF & VC-S n=9, data are shown as mean ± SEM.
